# Glyceridic and Unsaponifiable Components of Microencapsulated Sacha Inchi (*Plukenetia huayllabambana* L. and *Plukenetia volubilis* L.) Edible Oils

**DOI:** 10.3390/foods8120671

**Published:** 2019-12-12

**Authors:** Nancy A. Chasquibol, Gabriela Gallardo, Raquel B. Gómez-Coca, Diego Trujillo, Wenceslao Moreda, M. Carmen Pérez-Camino

**Affiliations:** 1 Center of Studies and Innovation of Functional Foods (CEIAF)-Faculty of Industrial Engineering, Institute of Scientific Research, IDIC, University of Lima, Avda. Javier Prado Este, 4600 Surco, Lima 15023, Peru; Nchasquibol@ulima.edu.pe; 2 National Institute of Industrial Technology, INTI- Av. Gral. Paz 5445, San Martín, Buenos Aires B1650WAB, Argentina; ggallar@inti.gob.ar; 3 Department of Characterization and Quality of Lipids, Instituto de la Grasa-CSIC, Ctra. Sevilla-Utrera km 1, Campus University Pablo de Olavide. Bg. 46, E-41013 Sevilla, Spain; raquel.coca@ig.csic.es (R.B.G.-C.); dtg.7@hotmail.com (D.T.); wmoreda@ig.csic.es (W.M.)

**Keywords:** analytical methods, coating materials, microencapsulation, omega-3, phytosterols, sacha inchi oils, tocopherols

## Abstract

Sacha inchi (*Plukenetia huayllabambana* L. and *Plukenetia volubilis* L.) edible oils were microencapsulated and the lipid fraction of the microparticles was characterized. Hi-cap^®^, Capsule^®^, Arabic gum, and the binary combination of Arabic gum + maltodextrin and the ternary combination of Arabic gum + maltodextrin + whey protein isolate, were used as coating materials for the encapsulation process using spray-drying. The surface and the total oils obtained from the microparticles were evaluated in terms of fatty acid composition, minor glyceride polar compounds, polymers, oxidized triglycerides, diglycerides, monoglycerides, and free fatty acids, along with their unsaponifiable components, sterols, and tocopherols. Differences between the original oils and the microencapsulated ones were determined. The most remarkable results included the presence of polymers when there were none in the original oils, the slight loss in ω3-fatty acids, up to 6%, the loss in tocopherols, in some of the cases around 30%, the maintaining of the phytosterol in their initial levels and the presence of cholesterol in the oils encapsulated with whey protein isolate.

## 1. Introduction

Since its emergence in the thirties, the microencapsulation process continues as an innovative technique for use in very different fields to those for which it was originally developed. The pharmaceutical industry made its first industrial developments by launching promising new applications that have been growing ever since with the appearance of new materials and processes. The food industry has also found a great tool in microencapsulation for solving problems, which are mainly related to preservation, even though in this field the advances have not turned out to be as spectacular as they have been in other areas. Actually, when it comes to food microencapsulation the main objectives are the protection of active principles, essential oils, and flavor so that they can be available with all their properties for a longer period of time [1]. In the particular case of polyunsaturated oils, although with some limitations related to shelf life, depending on the coating material, the encapsulation process helps to slow down the oxidation process [2]. Currently we can find many suitable dairy preparations for infants, which contain EPA (eicosapentaenoic acid) and DHA (docosahexaenoic acid) with no fishy taste or rancidity problems during their sell-by date. Walnut oil is another polyunsaturated oil that has been successfully microencapsulated using maltodextrin combined with hydroxypropyl methylcellulose as wall materials and spray-drying [3]. Carneiro et al. [4] encapsulated linseed oils with four types of wall materials in order to determine how to maximize the encapsulation efficiency and to minimize lipid oxidation. Gallardo et al. [5] also microencapsulated linseed oil by spray-drying and applied it to bread manufacturing. Microencapsulated ω3-fatty acids have been incorporated into many other food preparations such as chocolate, powdered drink mixes, instant coffees, and teas, increasing the bioavailability of ω3-fatty acids and increasing its original shelf life [6]. Recently, promising valuable manuscripts on chia oil (>60% linolenic acid) encapsulations have been published [7,8,9,10,11,12].

The sacha inchi seeds *Plukenetia huayllabambana* L. and the most commonly commercialized variety *Plukenetia volubilis* L. yield highly appreciated edible oils with a special green-fruit smell and a particularly high content in polyunsaturated fatty acids. The fatty acid composition is considered of great importance for human health as it is linked to the prevention of cardiovascular diseases and high blood pressure. ω-3-linolenic acid (Ln) is the main fatty acid in both sacha inchi oil varieties, with percentages over 51% and 48% in *P*. *huayllabambana* and *P*. *volubilis,* respectively, followed by α-linoleic (L) and oleic (O) acids [13,14,15,16]. This special fatty acid composition makes these oils very vulnerable to oxidation, developing a rancid smell, which affects the organoleptic characteristics of the foods containing them. In this context, sacha inchi oils (SIOs) are good candidates to be processed with the aid of this technique in order to add them to food preparations while keeping their highly valuable compositions. Thus, at the beginning of this decade the first research on the microencapsulation process for the SIOs appeared and the research and publications continue to date [17,18,19,20].

Maltodextrin (MD) and/or Arabic gum (AG) are the most widely used coating materials. However, new ones like corn zein [17], whey protein isolate (WPI) [21], ovalbumin, pectin, and xanthan gum [18] are being tested. Regarding the formation of the final microencapsulated products, in the particular case of the studies with sacha inchi oils, spray-drying is the preferred system over other types of drying [20]. A freeze-drying system is also used [22]. Recently the use of spray drying and spray chilling technologies were combined to obtain microparticles loaded with sacha inchi oil with a double shell [23]. Da Silva Soares et al. [24] presented the characterization of microparticles of sacha inchi *P*. *volubilis* oil obtained by the complex coacervation of ovalbumin and sodium alginate.

Most of the research carried out on microencapsulation focuses on the process itself and the search for the best materials. The physical properties of the microparticles obtained are thoroughly studied with the latest and most precise techniques, as well as their oxidative stability. However, few papers address the issue of how this process affects the oil itself, or its major and minor components [25]. What is more, only a small number of research groups address the encapsulation process from the point of view of the oil components alone [26,27,28,29].

The aim of this study was to evaluate how the encapsulation process affects the ω-3-fatty acid composition and the minor glyceridic and unsaponifiable components of virgin sacha inchi, *P. huayllabambana* and *P. volubilis*, oils (SIHO and SIVO, respectively) once they have been microencapsulated by spray-drying using different coating materials.

## 2. Materials and Methods

### 2.1. Raw Material

The sacha inchi seeds (*P. huayllabambana* L. and *P*. *volubilis* L. varieties) were collected in the Rodriguez de Mendoza area (Peru). The oils from the two varieties (SIHO and SIVO) were obtained by a cold-press system in a pilot plant in the Center of Studies and Innovation of Functional Foods (CEIAF) from Lima University. They were kept at 4 °C in the dark until use.

The coating materials, MD, AG, and WPI were obtained from Frutarom-Perú S.A. Also, two chemically n-octenyl succinic andydrid (OSAN)-modified starches, Capsul^®^ TA (derived from Tapioca starch, (Capsul)) and Hi-cap^®^ 100 TM (derived from waxy maize, (Hi-cap)) were tested, both (Capsul and Hi-cap) supplied by Ingredion. These wall materials were chosen based on their availability in the study area, their price, and their efficiency in other similar processes.

### 2.2. Solvents and Reactants

Ethanol, diethyl ether, hexane, chloroform, anhydrous sodium sulphate, potassium hydroxide, sulphuric acid, and ammonium hydroxide, were of analytical grade and were purchased from Merck-Spain (Merck Group, Darmstadt, Germany). Silica cartridges for solid-phase extraction (SPE 6 cc/1 g) were from Varian (EA Middelburg, The Netherlands). Tetrahydrofuran (HPLC grade) was supplied by VWR International (West Chester, PA, USA). Pyridine was from Sigma-Aldrich and BSTFA-TMCS (N,O-Bis (trimethylsilyl) trifluoroacetamide-Trimethylsilyl chloride) (99:1, *v/v*) a derivatizing reagent for GC was from TCI, Co., LTD, Tokyo, Japan.

### 2.3. Preparation of Sacha Inchi Oil Microparticles by Spray-Drying

The microencapsulation experiments were carried out using five coating materials with each sacha inchi oil ecotype.

For SIHO the samples were: 1. SIHO + Hi-cap, 2. SIHO + Capsul, 3. SIHO + AG, 4. Binary blend: SIHO + AG + MD, and 5. Ternary blend: SIHO + AG-MD + WPI.

For SIVO the samples were: 1. SIVO + Hi-cap, 2. SIVO + Capsul, 3. SIVO + AG, 4. Binary blend: SIVO + AG + MD, and 5. Ternary blend: SIVO + AG-MD + WPI.

Solutions were prepared with the coating materials and distilled water according to specifications given by the provider: MD, Capsul, and Hi-cap were kept at the controlled temperature of 25 °C until completely dissolved. For AG, the mixture was stirred overnight and for WPI the mixture was heated at 80 °C in order to partially denature the protein and open the globular chains. The total solid concentration was fixed at 27%. Sacha inchi oil was then added to the coating material solution at a concentration of 20% with respect to total solids. Emulsions were formed using a Silverson homogenizer L5M-A operating at 9000 rpm for 10 min at a very low temperature. The resultant mixture was used in the spray-drying procedure.

The spray-drying process was carried out by a laboratory scale spray-dryer (mini spray-dryer Büchi, B-290 Switzerland with a nozzle atomization system of 0.7 mm diameter) operated at an air inlet and outlet temperature of 150 and 80 °C, respectively, and a flow rate of 55 mL·min^−1^, fed by a peristaltic pump operating at a flow rate of 12 mL·min^−1^. The dried powders collected were stored in opaque hermetic bags at −15 °C for further analysis. 

### 2.4. Extraction of the Surface and Total Oil

From the dried powders, the surface oil (SO) and total oil (TO; oil trapped inside the microparticle core plus surface oil) were obtained in order to study their compositions. The SO is the oil which did not react with the solid matrix and can be easily obtained via organic solvents. Thus, in the spray-dried powder, the SO was determined gravimetrically by immersing an aliquot into hexane (1:10 *w/v*) for ten minutes at room temperature. Next, the solution was filtered through a Whatman no. 1 filter paper containing anhydrous sodium sulphate and evaporated using a rotary evaporator until dryness followed by a soft nitrogen stream until constant weight [30]. The data of the SO and the sacha inchi oil added (SIO) for encapsulation, served to determine the efficacy of the encapsulation (EE) by using the equation: EE (%) = (SIO − SO) × 100/SIO. Where SIO is the oil used for the encapsulation process and SO the surface oil obtained in the aliquot of the microparticles.

The TO of the microparticle samples were extracted through a basic digestion following a standardized procedure [31] with slight modifications: an aliquot of 5 ± 0.01 g of microparticles was added to 40 mL of deionized water heated at 65 °C. After stirring, 8 mL of 30% NH_4_OH were added and the admixture was stirred at 65 °C for 15 min. After cooling at room temperature, the lipids were extracted by three liquid–liquid extractions with the following sequence of solvents: a first extraction with ethanol (20 mL) followed by diethyl ether (50 mL) and then hexane (50 mL); a second extraction with diethyl ether (25 mL), hexane (25 mL), and ethanol (10 mL); and a third extraction with diethyl ether (25 mL) and hexane (25 mL). If some emulsions appeared 10 mL of ethanol were used. The extracts were filtered through a Whatman n. 1 filter paper containing anhydrous Na_2_SO_4_, and subsequently taken to a rotary evaporator to remove the solvent. Next, the samples were brought to constant weight using a stream of nitrogen.

### 2.5. Analytical Determinations

The physicochemical characteristics of the microparticles, particle size distribution and morphology, along with oxidative stability and their shelf life at 25 °C were already described [21].

In the present work, we focused on the chemical changes that the whole microencapsulation variable process (humidity, temperature, oxygen exposition, and wall materials) would produce on the oil components of the microparticles, determining the SO and the TO.

The initial SIOs (SIHO and SIVO) and the oils extracted from the microparticles (the SO and the TO) were characterized by the determinations detailed below: fatty acid composition, determined as fatty acid methyl esters (FAMEs); glyceridic polar compounds, namely triglycerides polymers (TG-P), oxidized triglycerides (ox-TG), diglycerides (DG), monoglycerides (MG), free fatty acids (FFA); and two of the main components of the unsaponifiable matter (tocopherols and sterols).

#### 2.5.1. Fatty Acid Composition Analysis

FAMEs were prepared according to the IUPAC Standard Method [32]. The oil samples in hexane (50 mg·2 mL^−1^) were transesterified using 250 µL KOH/MeOH 2 N and the FAMEs formed were analyzed using a 7890B Agilent gas chromatograph (Agilent Technologies, USA) equipped with a SP2380 polar capillary column (poly (90% biscyanopropyl–10% cyanopropylphenyl-siloxane, 60 m × 0.25 mm i.d.; 0.20 μm film thickness) and a flame ionization detector (FID). The injector and detector temperatures were maintained at 225 and 250 °C, respectively. Hydrogen was used as carrier gas at a flow rate of 1.0 mL·min^−1^. The oven temperature was set at 165 °C and increased to 230 °C at 3 °C·min^−1^ maintaining this temperature for 2 minutes. The injection volume was 1 μL. 

#### 2.5.2. Minor Glyceride Polar Compounds Analysis

The total quantity and the distribution of the minor glyceride polar compounds in the original SIOs and in the microencapsulated, both the superficial and the total one, were determined by high performance size exclusion chromatography (HPSEC) [26,28]. Previously, the oil fraction to be quantified was isolated using silica-solid phase extraction (Si-SPE). One hundred milligrams of the oil sample were weighed with mg precision and introduced into the Si-SPE column (previously conditioned with 6 mL hexane) using hexane (1 mL × 3 times) and separated into two fractions. The first fraction [unaltered triacylglycerols (TG)] was eluted with 15 mL of an hexane:diethyl ether (87:13, *v/v*) mixture and discarded. Subsequently, a second fraction (containing minor glyceride polar compounds) was eluted with 15 mL diethyl ether, which, after evaporation until dryness, was re-dissolved with 1 mL tetrahydrofuran (THF).

The separation and quantification of the individual glyceride polar compounds were performed by HPSEC using a chromatograph equipped with a HP1100 pump (Hewlett-Packard 1050 series), a Rheodyne 7725i injector (10 μL sample loop), and a refractive index detector (HP 1037 A, Agilent Technologies, Palo Alto, CA, USA). The separation was performed on a 100 Å column (25 cm × 0.77 cm i.d.) packed with porous, highly cross-linked styrene-divinylbenzene copolymers (5 μm film thickness; Agilent Technologies, Palo Alto, CA, USA). THF was used as the mobile phase at a flow rate of 0.7 mL·min^−1^. Quantitation of individual and total polar compounds was achieved using an external standard of pure TG in THF at concentrations of 0.1–2 mg·mL^−1^.

#### 2.5.3. Tocopherol Analysis

Tocopherols were determined according to the IUPAC Standard Method 2432 [33]. Oil solutions in hexane at concentrations of 5 mg·mL^−1^ were prepared and analyzed by HPLC fitted with a Si-column (250 mm × 4 mm i.d.; 4 μm particle size). The elution solvent was a mixture of hexane:2-propanol (99:1, *v/v*) at a flow rate of 1 mL·min^−1^. Detection was done by fluorescence (RF-10AXL Shimadzu fluorescence detector, Shimadzu, Columbia, MD, USA), setting excitation and emission at 290 and 330 nm wave lengths, respectively. For quantitative determinations, standards of tocopherols in hexane at concentrations of 4–6 μg·mL^−1^ were prepared and injected.

#### 2.5.4. Sterol Composition Analysis

A faster analytical method for the determination of phytosterols in small amounts of seed oils was applied [34]. This new procedure was followed with slight modifications mainly concerning the separation by an aminopropyl cartridge, a step, which was avoided in our case. Briefly: 50 mg of the oil samples, (SIOs, the surface ones, and total ones), were weighed with mg precision in a 20 mL test tube with screw cap and 10 µL of the internal standard of α-cholestanol (1 mg·mL^−1^ in chloroform) were added. The solvent was evaporated under a nitrogen steam. Subsequently, 5 mL of MeONa (3% *wt*/*v*) were added and the tube was placed in a bath at 80 °C for 30 min. Next, 5 mL of the admixture H_2_SO_4_ in MeOH (4% *v/v*) were added and maintained for 30 min at the same temperature. The lipid fraction was extracted three times with hexane (2 mL each), washed two times with water (2 mL each), and treated with anhydrous sodium sulfate to eliminate traces of water. The solution was evaporated until dryness under nitrogen, derivatized with 500 μL of the silylating reagent: BSTFA-TMCS:Pyridine1:1 *(v/v*) and analyzed by GC. The gas chromatograph (Agilent 6890N, Palo Alto, CA, USA) was equipped with a fused silica low-polarity capillary column (poly (5% diphenyl−95% dimethyl) siloxane, 30 m × 0.25 mm i.d. × 0.25 μm film thickness), and FID. The oven program was isothermally set at 260 °C with a 1:50 split ratio. Hydrogen was used as carrier gas at a 1 mL·min^−1^ flow rate. The temperature of the injector and detector was 300 °C. 

### 2.6. Data Analysis 

All the data were the average of three determinations that were statistically analyzed using the software Statistica 8.0 (StatSoft). Mean analysis was performed using Duncan’s procedure at *p* ≤ 0.05. 

## 3. Results and Discussion

Since the SIOs are considered a good source of essential fatty acids, (α-L and ω3-Ln), it would be of great interest to incorporate them into food products, such as bakery products, energy drinks, infant formulations, snacks, etc. Therefore, it becomes necessary to preserve them on the one hand, and on the other to facilitate their inclusion as food ingredients. A good way to achieve this is to do it microencapsulated form. So, their fatty acid composition could remain intact for a longer period of time and, it would also ensure that the product does not evolve towards notes of rancidity that may be rejected by consumers. In this way, it is necessary to know the most appropriate coating materials and how the microencapsulation process affects the oil composition, in terms of both changes in the ω3-fatty acid and also the minor components of high value such as sterols and tocopherols.

The obtaining of the SO by immersing the microparticle samples in hexane and the TO by alkaline digestion were done with special care, avoiding high temperatures during the evaporation of solvents. Exposure to air was also avoided by protecting it under nitrogen flow to guarantee that the changes were due to the encapsulation process and not to the process of obtaining the oil samples.

According to Table 1, the SO percentages were between 0.5% and 7.7% for SIHO and between 0.7% and 7.6% for SIVO. For both oils the wall materials Capsul and Hi-cap presented lower surface oil and consequently a higher encapsulation efficiency (EE). On the contrary, the ternary blend AG + MD + WPI gave the highest surface oil and consequently the lowest EE for both oils. 

These data cannot be due to or explained by the physical size of the particles, since, for both SIOs, the particles with the ternary blend (AG + MD + WPI) presented the largest sizes (6.1–9.8 µm for SIHO and SIVO, respectively) [21] and, consequently, the lowest surface/volume ratio. On the contrary, Capsul, also for both SIOs, was the wall material with the smallest particle size (2.5 and 1.2 for SIHO and SIVO, respectively, data not included in the above-mentioned paper) showing the majority of the population collapsed structures.

### 3.1. Fatty Acid Composition

The fatty acids detected in the samples of SIOs under study comprised the saturated group, which included the acids C16:0, C17:0, C18:0, and C20:0; the monounsaturated acids determined were C16:1ω9, C16:1ω7, C17:1, C18:1ω9, C18:1ω7, and C20:1ω9; and the polyunsaturated acids were C18:2ω6 and C18:3ω3. Τhe data of the fatty acid composition of the initial SIOs are included in Table 2, where the differences between the two sacha inchi oil varieties can be observed. The oils from *P. huayllabambana* showed a higher unsaturation level than those from *P. volubilis*, having more than half its fatty acids formed by ω3-linolenic acid (58.12%) followed by ω6-linoleic (>25%) and ω9-oleic (7.95%). These data are in agreement with the requirement of the technical regulation of Peru (NTP) for sacha inchi oils of the *P. huayllabambana* variety, where minimum levels of oleic (7.9%), linoleic (24.0%) and linolenic (55.0%) acids are required [35]. In relation to *P. volubilis*, the results of the fatty acid composition obtained for this oil were also in agreement with the requirements of NTP for this variety (oleic acid >8.9%, linoleic acid >32.1% and linolenic acid >44.7%). Many researchers consider a diet healthy when it contains a lipid fraction rich in ω3-fatty acids [36]. Thus, a lower ratio of ω6-/ω3-fatty acids would be desirable in order to reduce the risk of many of the chronic diseases of high prevalence in developing countries [37]. The exact value for the ω6/ω3-fatty acid ratio is given in many papers, but certain studies indicate that the optimal ratio may vary depending on the disease under consideration.

After the extraction of the microencapsulated oils, both from the surface and from the total microparticles, in the case of the *P. huayllabambana* variety (Table 2) a slight reduction in the Ln acid percentages was observed (mainly in the total oil fraction) compared to the initial oil. Thus, the encapsulation of *P. huayllabambana* with the ternary coating material (AG + MD + WPI) showed a value of 54.50% for Ln, which represented a 6.2% loss during the encapsulation procedure. The rest of the coating material losses during the encapsulation process varied between 4.7% and 0%.

In the case of the surface or non-encapsulated oil the loss was only appreciated when the encapsulation was done with AG as coating material (5%). In the oils obtained from the particles done with the *P. volubilis* variety, the decrease in the Ln percentage was not so evident, although the highest losses (around 3%) were observed for the surface oil encapsulated with Capsul as wall material.

Ln is the major and most abundant unsaturated acid in these oils and the first to be altered in the thermal conditions used in the spray-dryer equipment. Although the maximum temperature was not excessively high and the time was short, it may be that during the drying processes there was a greater shear effect, which makes the maximum temperature at some points higher than the average indicated during the passage through the spry-dryer. Under these conditions Ln is the acid most likely to be altered. It is important to note that concentrations below 1% for minor fatty acids, saturated or unsaturated, (C16:1, C17:0, and C17:1) were also possible to quantify in the encapsulated samples, and it was therefore deduced that they were not lost during the encapsulation process.

Regarding the *trans* fatty acid isomers (from oleic, linoleic, and linolenic acids), it is well known that they are produced from their *cis*-unsaturated counterparts during oil exposure at high temperatures as well as during the hydrogenation process in the presence of hydrogen and metal-catalysts [38]. The quantification can be done in the same chromatogram and conditions as for the rest of the fatty acids since they are eluted just before their corresponding *cis*-isomers. After the study in detail of the fatty acid chromatograms of all of the samples, the initial and the encapsulated oils, surface, or total ones, no peaks corresponding to the presence of *trans* fatty acids were present. That meant that during the whole encapsulation process, including the spray-drying stage, isomerization phenomena did not take place. Several papers have focused on the encapsulation feasibility of a very specific group of polyunsaturated fatty acids, conjugated linoleic acid (CLA). CLA contains a very high proportion of *trans*-fatty acid isomers with the C10-*trans*-C12-*cis* isomer often being the most prevalent [27,39,40] and, no report has highlighted an increase in the amount of the *trans-*isomers. What is more, it was pointed out that the composition of the *trans*-CLA isomer proportion was reduced significantly in microencapsulated oil samples with respect to the initial oil samples with the consequent increase in the saturated fatty acids, as each fatty acid methyl ester is expressed as a percentage on the total ones [27].

### 3.2. Minor Glyceride Polar Compound Composition

**Figure 1:**
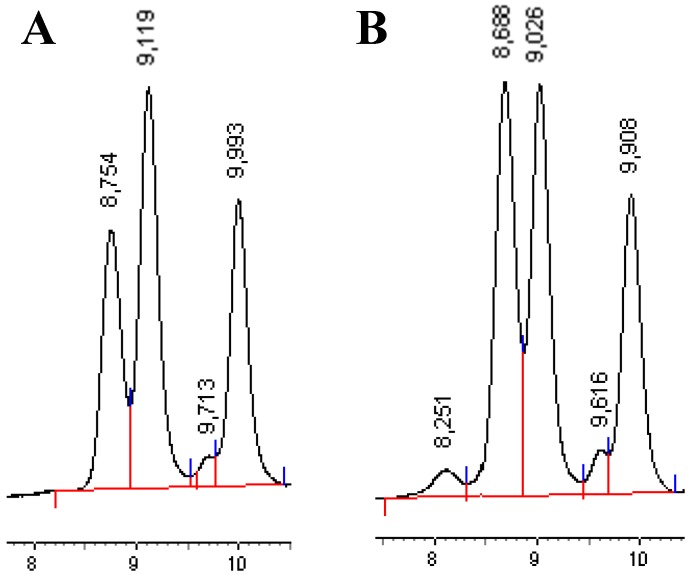
High performance size exclusion (HPSE) chromatograms of minor glyceride polar compounds from. (**A**) SIHO and (**B**) total oil from microencapsulated SIHO. Rt ~ 8.2 TG-P; Rt ~ 8.7 ox-TG; Rt ~ 9.1 DG; Rt ~ 9.7–9.6 MG; Rt ~ 9.9 FFA.

The so-called minor glyceride polar compounds comprise several families of compounds of very different molecular size, which is why they can be separated easily and quantified by HPSEC. TG-P, oxTG, DG, MG, and FFA elute from the chromatographic column in the opposite order of their molecular size. Those compounds correspond to three main alterations, which the oils exposed to high temperature suffer, atmospheric oxygen, and humidity. The corresponding alterations are thermic, oxidative, and hydrolytic, and can take place even during the oil extraction processes. These compounds were quantified by HPSEC with a refractive index detector using pure TGs as external standards and assuming the same response factor for all of them. Figure 1 includes two HPSE-chromatograms corresponding to one of the initial oils and to the total oil obtained after encapsulation. As can be observed, ox-TG, DG, MG, and FFA were detected in the original oils while the same ones, together with TG-P, were present in the encapsulated ones. Table 3 shows the data for the results of the thermal alteration (TG-P) in the first column, the oxidative alterations (ox-TG) in the second one, and diglycerides in the third one. Monoglycerides and free fatty acids were added to have data for the hydrolytic alteration (DG + MG + FA). The last column corresponds to total glyceride polar compounds (TPC) as the sum of the three alterations. The TPC values were very low, at 2.6% and 2.3% for the initial *P. huayllabambana* and *P. volubilis* oils, and it was in agreement with data obtained for any good edible oil [41]. For the encapsulated samples the TPC showed significant differences with their original oils, with the highest values (45 mg·g^−1^) being those obtained for the oils encapsulated with AG.

As can be observed in Figure 1A, four peaks appeared corresponding to oxTG, DG, MG, and FFA for the two sacha inchi oils under study. There was no presence of TG-P (retention time 8, in the original oil) meaning that during their extraction only a cold temperature was used and that they actually were crude or virgin oils. The major polar compounds for the two oil varieties corresponded to the hydrolytic alteration, which was 18.2 mg·g^−1^ oil for SIHO and 16.5 mg·g^−1^ for SIVO. After the encapsulation process one of the most remarkable results was the presence of TG-P (retention time ~8.2, Figure 1B) in all the oil samples (Table 3), which means that the temperature used in the step of spray-drying was sufficient for initiating the polymerization of the oils. HPSEC has been successfully used to carry out studies on microencapsulated oil [29]. Nevertheless, it was not specifically highlighted that polymers were formed during the encapsulation process. This aspect is not so obvious, since the investigations conducted with refined oils that already contained TG dimers or polymers originated during the deodorization stage of the refining process. Far from comparing this process with what happens in an oil refining process, microencapsulation will bring important benefits but also oil degradation. Thereby, the obtained results indicated that by starting from oils without polymers in their composition, polymerization took place and TG-P were present in both oil fractions, the encapsulated and the non-encapsulated ones, regardless of the coating material since its formation is closely related to temperature. The values for TG-P were in the range of 0.6–5.5 mg·g^−1^ oil, for *P. huayllabambana* and 0.2–0.9 mg·g^−1^ oil, for *P. volubilis*. Differences were observed for total and surface oils in both of them, with the total being slightly more polymerized than the surface ones. In relation to oxidative degradations (ox-TG), the values for the initial oils were 7.8 and 6.5 mg·g^−1^ (SIHO vs. SIVO). After encapsulation, these compounds experimented an increase in their values and for all the core materials with statistical significance with respect to their initial oils. Statistical differences were also obtained between surface and encapsulated oil fractions in total polar compounds, being slightly higher in the former for both types of oils. Working with dried microencapsulated fish oils, Velasco et al. [42] established important differences in the oxidation between free and encapsulated oil fractions for the first time. In their work, the authors highlighted the differences in the heterogeneous aspects of lipid oxidation in dried microencapsulated oils.

In relation to hydrolysis (DG + MG + FA), no differences were observed among the encapsulation materials, but somehow it was higher than in the initial oils. 

### 3.3. Tocopherol Composition

Tocopherols are ones of the most powerful natural antioxidants used as food additives [43]. Sacha inchi oil is the vegetable oil with one of the highest tocopherol contents, with γ- and δ-tocopherols being the main or unique species [14,44]. With respect to the regulations on this parameter, the NTP [35] included the value of 1900 mg·kg^−1^ as the minimum tocopherol content in sacha inchi oils, regardless of the variety.

**Figure 2:**
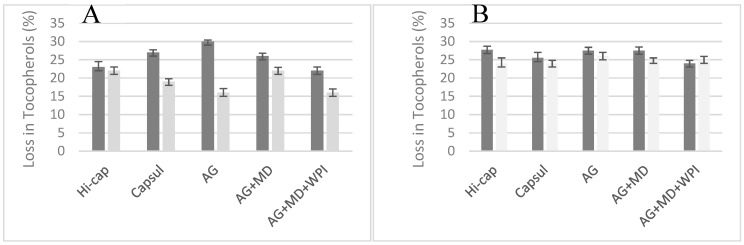
Loss in tocopherols (%) after the encapsulation processes: (**A**) *P. huayllabambana* oils and (**B**) *P. volubilis* oils. 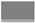 Total oil and 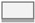 Surface oil.

In the oils under study, the total tocopherol concentrations were 2660 and 4393 mg·kg^−1^ for *P. huayllabambana* and *P. volubilis*, respectively (Table 4). They showed a γ/δ-tocopherol ratio of 2.1 and 1.7 for SIHO and SIVO, respectively. Figure 2A,B showed that after the encapsulation process the oils suffered a loss of 15–30% in the total tocopherol concentration for the surface and total oils and with respect to the original samples of SIHO. SIVO losses with respect to the original oil were in the range of 28–24% for total and surface oils, respectively. The data included in Table 4 indicate that the losses were mainly due in γ-tocopherol so that the γ/δ-tocopherol ratio decreased after encapsulation. For *P. huayllabambana* the ratio medium value after the encapsulation process was 1.6 in both fractions, whereas it was 1.0 for *P. volubilis*, clearly indicating that the γ-tocopherol losses were in higher proportion than δ-tocopherol for both oils and that such effect was more intense for SIVO than for SIHO. The higher stability of δ-tocopherol with respect to γ-tocopherol is in agreement with a recently published study where the stability of the different molecular species of tocopherols (α-, γ- and δ-) against continuous heating at different temperatures (100–180 °C) was evaluated [45]. The authors [45] concluded that δ-tocopherols were more stable than the γ- and α-species.

With respect to the coating materials, for both oil ecotypes the lower quantity of total tocopherols after encapsulation was determined when the process was done with AG for total oils. In relation to the surface oils, losses in tocopherols were lower than for the total oil mainly in the case of the *P. huayllabambana* variety where encapsulation with AG suffered the smallest loss with respect to the other coating materials. With regards to the data obtained for *P. volubilis* no differences were observed among the different materials.

### 3.4. Sterol Composition

Vegetable sterols or phytosterols are components of the unsaponifiable matter of vegetable oils with an important nutritional role and beneficial properties to human health. Several studies established that phytosterols have hypocholesterolemic effects, and decrease serum low-density lipoprotein (LDL) cholesterol levels by reducing intestinal cholesterol absorption [46,47]. As the levels of phytosterols present in food are not sufficient to achieve an effect on high cholesterol levels, some twenty years ago several commercial brands started to market products supplemented with phytosterols such as margarine, yogurt, or milk. However, the incorporation of phytosterols into other products processed under high heat was limited due to loss in functionality resulting from the deterioration caused by the processing conditions. These effects can be minimized through the use of microencapsulation as a strategy to protect and maintain phytosterol activity. In this sense, Alvim et al. [48] successfully proposed the use of a spray chilling technique for the production of microencapsulated lipids containing phytosterols. Spray-drying using AG and MD as wall materials have also been used to formulate phytosterol microparticles [49].

**Figure 3:**
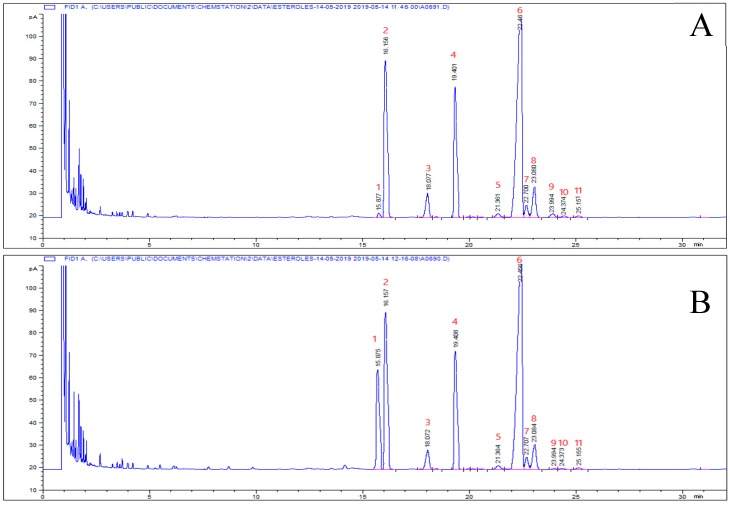
GC profile of sterols from SIHO. (**A**) Sterols of the original SIHO. (**B**) Sterols from the total oil of microencapsulated SIHO containing WPI as coating material. Peak numbers: (1): cholesterol; (2): internal standard (α-cholestanol); (3): campesterol; (4): stigmasterol; (5): clerosterol; (6): β-sitosterol; (7): sitostanol; (8): Δ5-avenasterol; (9): Δ5,24-stigmastadienol; (10): Δ7-stigmastenol; and (11): Δ7-avenasterol.

The three main sterols (>90%) found in SIOs were β-sitosterol (60%), stigmasterol (30%), and campesterol (5%; Figure 3A). Other minor sterols were also present: cholesterol (<0.4%), Δ7-campesterol (<1.0%), clerosterol (<1%), sitostanol (<1%), Δ5-avenasterol (1.2–2.0%), Δ5,24-stigmastadienol (<0.5%), Δ7-stigmastenol (<1%), and Δ7-avenasterol (0.2–3.4%). For this family of compounds no specification is included in the NTP regulation. Table 5 summarizes the main results obtained for the sterol determination including total sterols and β-sitosterol, as percentages of the total sterol contents, the sitostanol/campesterol ratio, and the cholesterol concentration in the initial oils and in those obtained from encapsulation, both from the surface and the total oils. Sacha inchi oils contain substantial amounts of total sterols (2056 and 2225 mg·kg^−1^ for SIHO and SIVO, respectively), with β-sitosterol as the major species (56.7% and 52.8%) as in all edible vegetable oils, followed by sitostanol and campesterol. Especially high sitostanol/campesterol ratios are typical of SIO and amounted to 5.9 and 3.7 for SIHO and SIVO, respectively.

After encapsulation, the average values obtained were: 2126 and 2312 mg·kg^−1^ for total extracted oil, around 4% higher but without significance. For the surface oil, the average sterol values were 1894 and 2271 mg·kg^−1^, which was 8% lower than the initial *P. huayllabambana* oil and remained at almost the same value for *P. volubilis.* In any case, the differences were statistically significant. In all samples of original oils and encapsulated ones, cholesterol did not reach a considerable value (around 0.2% of total sterols) except for the samples encapsulated with the ternary mixture of coating material. For total oil (not for the surface one) cholesterol concentrations of 1014 and 1072 mg·kg^−1^ were quantified for *P. huayllabambana* and *P. volubilis*, respectively, in samples encapsulated with whey protein isolate, clearly indicating that the cholesterol found comes from the coating material (Figure 3B, Table 5).

Despite the great interest in the importance of phytosterols and the need for it to be available for use in many processes, there is still little research verifying the changes suffered by the sterols coming from edible oils after encapsulation [25]. Recently Hue et al. [50] studied some physiochemical properties of kenaf seed oil microcapsules and determined their sterol compositions. They studied the evolution of phytosterols during accelerated storage conditions (from day 0 to day 24) but changes between the initial oils and the oils obtained after the process are not discussed [50].

## 4. Conclusions

The encapsulation process itself alters the oil, although to a minimum extent. The major fatty acid, ω3-Ln, suffered a loss of 6.2% during the encapsulation process of SIHO (for total oil) with the ternary coating material (AG + MD + WPI). In the case of SIVO, with a lower unsaturation level, the loss was lower (3%) when Capsul was used as wall material. It is interesting to note the absence of formation of *trans* fatty acid isomers throughout the processes.

Regarding the minor glyceride polar components, the TG-P are absent from the original oils, as no heat was used for their extraction. In contrast, all the encapsulated oils showed quantified levels of TG-P. As expected, because of its higher unsaturation level, samples coming from SIHO presented higher TG-P levels (0.6–5.5 mg·g^−1^ oil) than those formed with SIVO (0.2–0.9 mg·g^−1^ oil).

The greatest losses in minor non-glyceride components were observed in the tocopherols, 30−15% for the total and surface SIHO, respectively. In the case of SIVO losses, they were also in the range of 28–24% for total and surface oils γ-tocopherol losses were higher than δ-tocopherol for both SIOs. For both varieties (SIHO and SIVO), the encapsulation process with AG presented the highest losses in total oil.

Regarding the sterols, the losses were not statistically significant in any cases. The most remarkable and unexpected result was the presence of cholesterol when the ternary blend (AG + MD + WPI) was employed as coating material, for both SIOs. This is clearly due to the WPI material because this sterol appears in the unique formulation, which contains WPI and WPI came from an animal source.

## Figures and Tables

**Table 1:** Surface oil (SO) obtained by immersion with hexane, and encapsulation efficiency (EE).

Oil	Wall Material	SO (%*w*/*w*)	EE (%)
*P. huayllabambana*	Hi-cap	1.3 ± 0.1 ^f^	93.3 ± 0.5 ^C^
	Capsul	0.5 ± 0.1 ^h^	97.6 ± 0.5 ^A^
	AG	5.4 ± 0.1 ^f^	73.0 ± 1.5 ^E^
	AG + MD	4.8 ± 0.1 ^e^	75.8 ± 0.9 ^D^
	AG+ MD + WPI	7.7 ± 0.1 ^a^	61.1 ± 0.6 ^G^
*P. volubilis*	Hi-cap	0.7 ± 0.1 ^g,h^	96.5 ± 0.7 ^A,B^
	Capsul	0.8 ± 0.1 ^g^	96.0 ± 0.7 ^B^
	AG	6.5 ± 0.1 ^b^	67.4 ± 0.5 ^F^
	AG + MD	6.0 ± 0.1 ^c^	67.9 ± 0.5 ^F^
	AG+ MD + WPI	7.6 ± 0.1 ^a^	61.7 ± 0.6 ^G^

Different letters, indicate significant difference values at *p* < 0.05 (*n* = 3; lower case letters for SO and capital letters for EE).

**Table 2:** Fatty acid composition of sacha inchi oils before and after the encapsulation process (total and superficial oils extracted from microencapsulation with five wall materials). Data are expressed as percentages of total fatty acids.

Samples	Fatty Acid Composition (%)										
	C_16:0_	C_16:1_	C_17:0_	C_17:1_	C_18:0_	C_18:1_	C_18:2_	C_18:3_	C_20:0_	C_20:1_	*Trans*
***P. huayllabambana***											
**Initial oil**	4.50 ± 0.24	0.07 ± 0.01	0.06 ± 0.01	0.04 ± 0.01	1.75 ± 0.05	7.95 ± 0.30	26.10 ± 0.50	58.12 ± 0.55 ^a^	0.31 ± 0.15	0.29 ± 0.15	nd *
**Total encapsulated oils**											
SIHO + Hi-cap	4.58 ± 0.23	0.05 ± 0.01	0.05 ± 0.01	0.04 ± 0.01	1.77 ± 0.09	7.88 ± 0.30	26.57 ± 0.50	58.61 ± 0.56 ^a^	0.31 ± 0.15	0.28 ± 0.15	nd *
SIHO + Capsul	4.82 ± 0.25	0.03 ± 0.01	0.04 ± 0.01	0.04 ± 0.01	1.84 ± 0.09	8.57 ± 0.30	26.56 ± 0.50	57.58 ± 0.52 ^a^	0.34 ± 0.15	0.30 ± 0.14	nd *
SIHO + AG	4.57 ± 0.25	0.06 ± 0.01	0.07 ± 0.01	0.04 ± 0.01	2.08 ± 0.08	8.62 ± 0.30	28.52 ± 0.50	55.46 ± 0.55 ^b^	0.30 ± 0.15	0.28 ± 0.14	nd *
SIHO + AG + MD	5.20 ± 0.23	0.03 ± 0.01	0.07 ± 0.01	0.05 ± 0.01	1.78 ± 0.09	11.08 ± 0.30	25.56 ± 0.52	55.60 ± 0.54 ^b^	0.30 ± 0.15	0.22 ± 0.15	nd *
SIHO + AG + MD + WPI	5.38 ± 0.24	0.04 ± 0.01	0.06 ± 0.01	0.04 ± 0.01	1.94 ± 0.09	12.38 ± 0.30	25.21 ± 0.53	54.50 ± 0.55 ^b^	0.31 ± 0.15	0.22 ± 0.14	nd *
**Surface oils**											
SIHO + Hi-cap	4.65 ± 0.24	0.07 ± 0.01	0.06 ± 0.02	0.04 ± 0.00	1.70 ± 0.09	7.85 ± 0.35	26.20 ± 0.51	58.02 ± 0.55 ^a^	0.31 ± 0.15	0.29 ± 0.15	nd *
SIHO + Capsul	4.55 ± 0.23	0.07 ± 0.02	0.06 ± 0.02	0.04 ± 0.00	1.75 ± 0.09	7.90 ± 0.36	26.40 ± 0.50	58.00 ± 0.56 ^a^	0.33 ± 0.16	0.29 ± 0.10	nd *
SIHO + AG	4.52 ± 0.23	0.09 ± 0.01	0.08 ± 0.02	0.04 ± 0.01	2.13 ± 0.09	8.35 ± 0.35	28.98 ± 0.50	55.29 ± 0.54 ^b^	0.33 ± 0.15	0.23 ± 0.11	nd *
SIHO+ AG + MD	4.65 ± 0.24	0.06 ± 0.01	0.07 ± 0.02	0.04 ± 0.01	1.73 ± 0.09	7.84 ± 0.35	26.41 ± 0.58	58.71 ± 0.55 ^a^	0.30 ± 0.14	0.23 ± 0.12	nd *
SIHO + AG + MD + WPI	4.72 ± 0.25	0.07 ± 0.01	0.10 ± 0.01	0.04 ± 0.01	1.83 ± 0.09	8.07 ± 0.40	26.41 ± 0.58	58.26 ± 0.54 ^a^	0.32 ± 0.14	0.22 ± 0.10	nd *
***P. volubilis***											
**Initial oil**	3.95 ± 0.21	0.06 ± 0.01	0.09 ± 0.01	0.05 ± 0.01	3.05 ± 0.15	9.90 ± 0.35	34.42 ± 0.58	47.88 ± 0.44 ^A,B^	0.32 ± 0.15	0.28 ± 0.15	nd *
**Total encapsulated oils**											
SIVO + Hi-cap	4.06 ± 0.24	0.06 ± 0.01	0.09 ± 0.01	0.05 ± 0.01	3.06 ± 0.16	10.34 ± 0.34	34.27 ± 0.58	47.66 ± 0.45 ^B^	0.33 ± 0.15	0.28 ± 0.15	nd *
SIVO + Capsul	4.43 ± 0.23	0.08 ± 0.02	0.09 ± 0.01	0.05 ± 0.01	3.07 ± 0.16	12.04 ± 0.36	33.52 ± 0.58	46.18 ± 0.45 ^D^	0.31 ± 0.15	0.28 ± 0.15	nd *
SIVO + AG	4.21 ± 0.23	0.10 ± 0.02	0.79 ± 0.01	0.05 ± 0.01	3.34 ± 0.16	9.85 ± 0.35	33.91 ± 0.58	47.18 ± 0.41 ^C^	0.39 ± 0.01	0.23 ± 0.15	nd *
SIVO + AG + MD	4.10 ± 0.23	0.06 ± 0.02	0.05 ± 0.01	0.11 ± 0.01	3.12 ± 0.16	10.03 ± 0.35	34.27 ± 0.58	47.77 ± 045 ^B^	0.32 ± 0.01	0.23 ± 0.15	nd *
SIV O+ AG + MD + WPI	4.67 ± 0.23	0.09 ± 0.02	0.09 ± 0.01	0.05 ± 0.01	3.16 ± 0.16	11.44 ± 0.36	33.55 ± 0.58	46.40 ± 0.46 ^C,D^	0.32 ± 0.01	0.28 ± 0.15	nd *
**Surface oils**											
SIVO + Hi-cap	4.09 ± 0.20	0.09 ± 0.02	0.09 ± 0.01	0.09 ± 0.01	3.06 ± 0.01	10.45 ± 0.29	34.33 ± 0.56	47.38 ± 0.44 ^B,C^	0.32 ± 0.15	0.27 ± 0.15	nd *
SIVO + Capsul	4.09 ± 0.20	0.09 ± 0.02	0.09 ± 0.01	0.09 ± 0.01	3.08 ± 0.01	9.42 ± 0.25	34.66 ± 0.56	48.06 ± 0.43 ^A,B^	0.31 ± 0.15	0.28 ± 0.15	nd *
SIVO + AG	3.99 ± 0.20	0.09 ± 0.02	0.09 ± 0.01	0.09 ± 0.01	3.04 ± 0.01	9.25 ± 0.25	34.73 ± 0.54	48.47 ± 0.45 ^A^	0.30 ± 0.16	0.21 ± 0.15	nd *
SIVO + AG + MD	3.95 ± 0.20	0.06 ± 0.02	0.09 ± 0.01	0.09 ± 0.01	3.05 ± 0.01	9.28 ± 0.25	34.70 ± 0.54	48.48 ± 0.44 ^A^	0.32 ± 0.15	0.21 ± 0.15	nd *
SIVO + AG + MD + WPI	4.18 ± 0.20	0.06 ± 0.01	0.09 ± 0.01	0.09 ± 0.01	3.10 ± 0.01	10.16 ± 0.29	34.22 ± 0.55	47.71 ± 0.45 ^A,B^	0.34 ± 0.15	0.28 ± 0.15	nd *

Different letters for the major fatty acid (ω3-Ln) indicate significant difference values at *p* < 0.05 (*n* = 3; lower case letters for *P. huayllabambana* oil (SIHO) and capital letters for *P*. *volubilis* oil (SIVO)).nd *: not detected.

**Table 3:** Polar compounds in total and superficial oils extracted from microencapsulated oils of sacha inchi (*P. huayllabambana* and *P. volubilis*) with five wall materials and in the initial oils used for encapsulation.

Samples	Minor Glyceride Polar Compounds (mg·g^−1^)			
	TG-P	ox-TG	DG + MG + FA	Total PC
***P*. *huayllabambana***				
**Initial Oil**	nd *	7.8 ± 0.14	18.2 ± 0.15	26.0 ± 0.21 ^i^
**Total Encapsulated Oils**				
SIHO + Hi-cap	1.1 ± 0.02	13.7 ± 0.11	19.1 ± 0.16	33.9 ± 0.26 ^f^
SIHO + Capsul	2.0 ± 0.03	11.7 ± 0.12	19.1 ± 0.15	32.8 ± 0.32 ^h^
SIHO + AG	5.5 ± 0.06	20.5 ± 0.15	19.1 ± 0.15	45.1 ± 0.34 ^a^
SIHO + AG + MD	4.4 ± 0.07	20.3 ± 0.26	19.3 ± 0.15	44.0 ± 0.36 ^a^
SIHO + AG + MD + WPI	3.0 ± 0.05	17.7 ± 0.12	19.1 ± 0.15	39.8 ± 0.31 ^c^
**Surface Oils**				
SIHO + Hi-cap	0.6 ± 0.01	18.2 ± 0.10	19.3 ± 0.15	38.1 ± 0.22 ^d^
SIHO + Capsul	0.6 ± 0.05	11.0 ± 0.14	19.4 ± 0.17	31.0 ± 0.30 ^g^
SIHO + AG	0.6 ± 0.05	25.1 ± 0.20	19.3 ± 0.17	45.0 ± 0.38 ^a^
SIHO + AG + MD	0.8 ± 0.03	22.1 ± 0.10	19.2 ± 0.14	42.1 ± 0.28 ^b^
SIHO + AG + MD + WPI	1.0 ± 0.03	17.0 ± 0.11	19.0 ± 0.16	37.0 ± 0.24 ^e^
***P. volubilis***				
**Initial Oil**	nd *	6.5± 0.10	16.5± 0.16	23.0 ± 0.26 ^G^
**Total encapsulated Oils**				
SIVO + Hi-cap	0.4 ± 0.26	8.3 ± 0.26	17.0 ± 0.14	25.7 ± 0.35 ^F^
SIVO + Capsul	0.5 ± 0.26	8.1 ± 0.20	17.4 ± 0.16	26.0 ± 0.28 ^F^
SIVO + AG	0.9 ± 0.26	11.9 ± 0.12	17.4 ± 0.15	30.2 ± 0.28 ^B,C^
SIVO + AG + MD	0.2 ± 0.26	10.5 ± 0.26	17.4 ± 0.16	28.1 ± 0.28 ^D^
SIVO + AG + MD + WPI	0.7 ± 0.26	8.8 ± 0.26	17.4 ± 0.13	26.9 ± 0.24 ^E^
**Surface Oils**				
SIVO + Hi-cap	0.3 ± 0.26	11.3 ± 0.26	17.3 ± 0.16	28.9 ± 0.30 ^D^
SIVO + Capsul	0.9 ± 0.26	12.6 ± 0.14	17.4 ± 0.17	30.9 ± 0.29 ^B^
SIVO + AG	0.4 ± 0.26	14.4 ± 0.26	17.4 ± 0.15	32.2 ± 0.30 ^A^
SIVO + AG + MD	0.6 ± 0.26	14.9 ± 0.26	17.4 ± 0.11	32.9 ± 0.32 ^A^
SIVO + AG + MD + WPI	0.5 ± 0.26	13.4 ± 0.26	17.1 ± 0.18	31.0 ± 0.29 ^B^

Different letters, for total PC, indicate significant difference values at *p* < 0.05 (*n* = 3). Lower case letters for SIHO and capital letters for SIVO. nd *: not detected.

**Table 4:** Tocopherols in total and superficial oils extracted from microencapsulated oils of sacha inchi (*P. huayllabambana* and *P. volubilis*) with five wall materials and in the initial oils used for encapsulation.

	Tocopherols (mg·kg^−1^)											
	Total Extracted Oil						Surface Oil					
	α-	β-	γ-	δ-	Total	Ratio γ/δ	α-	β-	γ-	δ-	Total	Ratio γ/δ
***P. huayllabambana***												
Initial oil	nd *	nd *	1811 ± 20	849 ± 15	2660 ± 45 ^a^	2.1						
SIHO + Hi-cap	nd *	nd *	1295 ± 22	746 ± 14	2041 ± 40 ^d^	1.7	nd *	nd *	1221 ± 18	837 ± 14	2058 ± 32 ^d^	1.5
SIHO + Capsul	nd *	nd *	1155 ± 25	762 ± 15	1917 ± 30 ^e^	1.5	nd *	nd *	1300 ± 17	840 ± 20	2140 ± 32 ^c^	1.5
SIHO + AG	nd *	nd *	1104 ± 24	760 ± 14	1864 ± 35 ^f^	1.5	nd *	nd *	1375 ± 19	848 ± 15	2223 ± 36 ^b^	1.6
SIHO + AG + MD	nd *	nd *	1204 ± 22	764 ± 14	1968 ± 35 ^e^	1.6	nd *	nd *	1200 ± 19	848 ± 15	2048 ± 31 ^d^	1.4
SIHO + AG + MD + WPI	nd *	nd *	1290 ± 23	749 ± 16	2039 ± 35 ^d^	1.7	nd *	nd *	1406 ± 18	842 ± 12	2248 ± 30 ^b^	1.7
***P. volubilis***												
Initial Oil	nd *	nd *	2767 ± 32	1626 ± 30	4393 ± 45 ^A^	1.7						
SIVO + Hi-cap	nd *	nd *	1596 ± 33	1585 ± 32	3181 ± 35 ^D^	1.0	nd *	nd *	1720 ± 31	1624 ± 30	3344 ± 35 ^B^	1.1
SIVO + Capsul	nd *	nd *	1699 ± 30	1558 ± 30	3257 ± 35 ^C^	1.1	nd *	nd *	1710 ± 31	1625 ± 35	3335 ± 30 ^B^	1.1
SIVO + AG	nd *	nd *	1550 ± 31	1615 ± 30	3165 ± 32 ^D^	1.0	nd *	nd *	1613 ± 33	1614 ± 32	3227 ± 33 ^C^	1.0
SIVO + AG + MD	nd *	nd *	1586 ± 30	1591 ± 30	3177 ± 33 ^D^	1.0	nd *	nd *	1675 ± 33	1614 ± 33	3289 ± 35 ^C^	1.0
SIVO + AG + MD+ WPI	nd *	nd *	1765 ± 32	1581 ± 30	3346 ± 35 ^B^	1.1	nd *	nd *	1657 ± 31	1622 ± 34	3279 ± 35 ^C^	1.0

For total tocopherols, different letters indicate significant difference values at *p* < 0.05 (lower case letters for SIHO and capital letters for SIVO). nd *: not detected.

**Table 5:** Sterol concentrations in initial oils and those extracted after microencapsulation.

		Sterols from Total Microencapsulated Oil				Sterols from the Surface Oil			
	Samples	β-sitosterol (Mayor Phytosterol) % on Total Phytosterols	Stigmasterol/ Campesterol Ratio	Cholesterol (mg·kg^−1^)	Total Phytosterols (mg·kg^−1^)	β-sitosterol (Mayor Phytosterol) % on Total Phytosterols)	Stigmasterol/ Campesterol Ratio	Cholesterol (mg·kg^−1^)	Total Phytosterols (mg·kg^−1^)
*P. huayllabambana*	Initial oil	56.7 ± 1.5	5.9 ± 0,6	*	2056 ± 97 ^a,b^				
	SIHO + Hi-cap	60.7 ± 3.0	5.1 ± 0.5	*	2119 ± 90 ^a^	62.2 ± 3.5	5.2 ± 0.6	*	1934 ± 75 ^b,c^
	SIHO + Capsul	61.8 ± 3.8	4.9 ± 0.5	*	2154 ± 95 ^a^	63.7 ± 3.5	5.3 ± 0.6	*	1888 ± 79 ^c^
	SIHO + AG	60.8 ± 3.1	5.2 ± 0.5	*	2101 ± 92 ^a^	61.6 ± 3.0	5.2 ± 0.5	*	1893 ± 80 ^c^
	SIHO + AG + MD	63.7 ± 6.0	5.1 ± 0.5	*	2050 ± 75 ^a^	61.2 ± 3.2	5.3 ± 0.5	*	1855 ± 80 ^c^
	SIHO + AG + MD + WPI	62.5 ± 4.5	4.3 ± 0.5	1014 ± 30	2104 ± 90 ^a^	61.5 ± 3.1	5.4 ± 0.6	*	1901 ± 75 ^c^
*P. volubilis*	Initial oil	52.8 ± 2.0	3.3 ± 0.6	*	2225 ± 65 ^A^				
	SIVO + Hi-cap	58.2 ± 2.5	3.7 ± 0.5	*	2255 ± 65 ^A^	56.0 ± 2.9	3.4 ± 0.5	*	2269 ± 85 ^A^
	SIVO + Capsul	58.6 ± 3.9	3.8 ± 0.5	*	2282 ± 60 ^A^	56.0 ± 2.9	3.4 ± 0.5	*	2290 ± 90 ^A^
	SIVO + AG	55.8 ± 3.0	4.1 ± 0.5	*	2356 ± 70 ^A^	56.5 ± 3.0	3.8 ± 0.5	*	2277 ± 95 ^A^
	SIVO + AG + MD	56.3 ± 3.4	3.1 ± 0.6	*	2348 ± 65 ^A^	55.8 ± 3.0	3.9 ± 0.5	*	2240 ± 95 ^A^
	SIVO + AG + MD + WPI	56.0 ± 3.2	3.0 ± 0.7	1072 ± 32	2321 ± 55 ^A^	55.8 ± 3.0	4.0 ± 0.6	*	2279 ± 95 ^A^

In each column different letters indicate significant difference values at *p* < 0.05 (lower case letters for SIHO and capital letters for SIVO). *: <0.4% on total sterols.
